# Benzodiazepines Withdrawal: Initial Outcomes and Long-Term Impact on Falls in a French Nursing Home

**DOI:** 10.3390/pharmacy6020030

**Published:** 2018-04-06

**Authors:** Hervé Javelot, Anne Marquis, Emilie Antoine-Bernard, Jean Grandidier, Luisa Weiner, Thierry Javelot, Bruno Michel

**Affiliations:** 1 Maison Hospitalière de Baccarat, 54120 Baccarat, France; mhbpharma@free.fr (A.M.); ergo@mhb-baccarat.fr (E.A.-B.); mhbmedical@free.fr (J.G.); 2 Service de Pharmacie Clinique, Etablissement Public de Santé Alsace Nord, 67173 Brumath, France; 3 Clinique Psychiatrique, Service de Psychiatrie II, Hôpital Civil, CHU de Strasbourg, 67091 Strasbourg, France; luisa.weiner@chru-strasbourg.fr; 4 Equipe de Liaison et de Soins en Addictologie, C.H. Saint Jean de Dieu, 69008 Lyon, France; t.javelot@clinique-le-sermay.fr; 5 Service Pharmacie, CHU de Strasbourg, Faculté de Pharmacie, EA7396, 67091 Strasbourg, France; bruno.michel@chru-strasbourg.fr

**Keywords:** older patients, benzodiazepine, withdrawal, fall, deprescription

## Abstract

Long-term use of benzodiazepines (BZDs) is known to induce tolerance and dependence, and increase the risk of falls-related injuries in older adults. We present a study carried out in a French nursing home that concerns the implementation of a BZD withdrawal program reassessed at one year. BZD deprescription was achieved by gradual cessation of doses. A secondary benefit of this program was assessed by comparing the number of falls among residents before and after withdrawal. The number of falls was recorded over a six-month period prior to the onset of withdrawal (T1) and then over a six-month period after reassessment at one year (T2). At the beginning, 31 (28.7%) of the patients were under BZD. Total deprescription was obtained for 11 patients. The number of falls per patient over the T1 period was not different between the two groups (future non-withdrawn and withdrawn patients in BZD): 2.1 ± 1.3 and 2.3 ± 0.6 falls per resident, respectively. Conversely, the number of falls per patient was significantly decreased in the population completely withdrawn in BZD between the T1 and T2 periods (2.3 ± 0.6 vs. 0.5 ± 0.2 falls, *p* = 0.01). The results show that BZD deprescription, through a gradual reduction of doses, is possible to achieve.

## 1. Introduction

The risk of benzodiazepines (BZDs) dependence has been known for a long time and is a real public health problem [1,2,3]. Beyond dependence, their chronic use in elderly patients is linked to a well-documented increased risk of falls and cognitive deterioration [4,5,6,7,8,9,10,11].

In 2007, due to the growing chronic BZD use in France, the French National Health Authority (HAS) established guidelines for BZD cessation in older patients (professional recommendations entitled “Procedures for stopping benzodiazepines and similar drugs in older patients”; approximately two million elderly consume anxiolytics, mostly BZDs, chronically in France [3,12]). On the basis of the recommendations, several BZD withdrawal protocols were proposed: (i) gradual cessation of doses, (ii) gradual cessation of doses accompanied by pharmacological treatment and finally (iii) gradual cessation doses associated with cognitive-behavioral therapy [3].

In September 2011, the Regional Health Agency (RHA) of Lorraine, France requested a reassessment of BZD prescriptions of all residents of nursing homes.

This study assessed the implementation of a one-year BZD withdrawal program and the number of associated patient falls.

## 2. Materials and Methods 

### 2.1. General Description of the Study and Informed Consent

This study was carried out between 2011 and 2013 in a nursing home (Maison Hospitalière de Baccarat-France), which had 108 residents. On the occasion of the monthly consultations in September 2011, the BZD withdrawal proposition was suggested to residents. Patients were informed about the BZD cessation program (oral and written transmission) and their consent to participate in the withdrawal challenge was requested (patient or primary caregiver). This request was reformulated during the subsequent monthly consultations on October, November and December 2011 (four-month program). 

### 2.2. BZD Withdrawal

The inclusion criterion for residents was to be treated by at least one BZD. The exclusion criterion was alcoholism or epilepsy. Consequently, withdrawal was proposed exclusively for patients receiving a BZD for symptomatic treatment of severe and/or disabling anxiety manifestations, excluding situations such as “prevention and treatment of delirium tremens and other alcohol withdrawal manifestations” and epilepsy treatment. 

Deprescribing of the BZD is not mandatory in France and the request of the Regional Health Agency (RHA) of Lorraine was an original and incentive approach to follow the recommendations of the HAS. The data about BZD withdrawal were collected within each nursing home (patient electronic medical record). 

Based on the different BZD withdrawal options proposed by the HAS [3], two withdrawal methods were potentially applicable in the nursing home: (i) gradual cessation of doses and (ii) gradual cessation of doses accompanied by pharmacological treatment. This latter option was not chosen by prescribers considering the two proposed pharmacological approaches: (i) the use of paroxetine or imipramine, substances having anticholinergic properties [3,13], and (ii) the use of buspirone, with no demonstrated efficacy for BZD withdrawal versus placebo [3,14].

The gradual cessation was based on the general recommendations of the HAS. These indicated (i) that it could be performed usually in 4–10 weeks or potentially over several months in the case of long-term or high-dose users, (ii) that an initial dose decrease of 25% in the first week was an example of reduction in the case of a 4–10 weeks cessation program [3]. Beyond these recommendations, more specific and pragmatic aspects guided the prescribers. 

The decreasing dosage steps were adjustable depending on the possibility of cutting the tablets. A gradual switch to intermediate and short half-life BZD was promoted for patients receiving a long or intermediate half-life BZD. Finally, the duration of the decreasing steps required for BZD deprescription were adjusted according to the clinical progression.

In practice, the physician and the pharmacist proposed BZD withdrawal to residents by exposing the benefit–risk ratio related to these molecules (oral transmission of a standardized information about risks associated with BZD—tolerance, dependence and risk of falls with injuries—and benefits associated with these drugs on anxiety or insomnia in short-term use). Program presentation was therefore based on a pharmacist–physician collaborative practice (binomial intervention in order to improve adherence). This proposition was reformulated in case of initial refusal of the patient during the four months of the program (short reminder about benefit–risk ratio related to BZD and opportunity of withdrawal from the program). 

At the end of the four months of program and at one year, an assessment was carried out. During these two periods of evaluation, the renewed prescriptions, the stopped prescriptions, the treatment declines (including the posology decrease, the treatment breaks if several BZDs or similar drugs were co-prescribed, the substitution of one BZD with another with shorter half-life) and possible increases in treatment (including the following cases: effective increases in dosage, addition of BZD or similar drug, substitution of one BZD by another with longer half-life) were investigated.

### 2.3. Evaluation of the Number of Falls

A fall is defined by the WHO (World Health Organization) as “*an event which results in a person coming to rest inadvertently on the ground or floor or other lower level*” [15]. This definition was used for the secondary purpose of the study. Falls’ records were routinely taken by the occupational therapist of the institution (these data were not a mandatory record associated with the reassessment of BZD prescriptions requested by the RHA of Lorraine). Fall reduction strategy (environmental management) had been previously achieved in the establishment and no further changes occurred during the BZD withdrawal program.

The number of falls was recorded over a six-month period prior to the onset of withdrawal (T1 period) and then over a six-month period after the reassessment of the one-year BZD prescriptions (T2 period). The number of falls among withdrawn patients in BZD [total withdrawal] was then compared to those without withdrawal. An analysis was carried out to check whether the populations were homogeneous regarding the number of falls before the implementation of the BZD deprescription program by a Student *t-*test on unmatched values. Subsequently, comparisons within the two populations between the pre- and post-withdrawal periods were performed by Student *t-*tests on the matched values.

## 3. Results

### 3.1. BZD Deprescription

On 1 September 2011, 31 (28.7%) patients were on BZD therapy: 26 women and 5 men; the mean age of patients was 89 years (approximately 20% with an age greater than or equal to 95 years). All 31 had BZD prescription for over a month and 27 for longer than six months. Of these 31 patients, 20 (64.5%) received intermediate or long half-life BZDs (alprazolam, lorazepam, bromazepam, clonazepam, diazepam and prazepam); 11 patients (35.5%) were taking BZD (or non-benzodiazepine “Z-drugs”: zolpidem and zopiclone) of short half-life (less than 10 h: oxazepam, zolpidem and zopiclone). A total BZD withdrawal was obtained in one year for 11 patients out of 31 (35.5%). The patients whose prescriptions could be reassessed at four months and at one year did not have their BZD treatment increased. It should be noted that the patients who were not withdrawn at four months corresponded to patients who either categorically or definitively refused BZD deprescription, or situations for which the practitioners judged that the clinical situation was not suitable for a withdrawal attempt. 

Table 1 shows the evolution of BZD deprescription practices at four-month and one-year reassessments. 

### 3.2. Evaluation of Number of Falls

**Figure 1:**
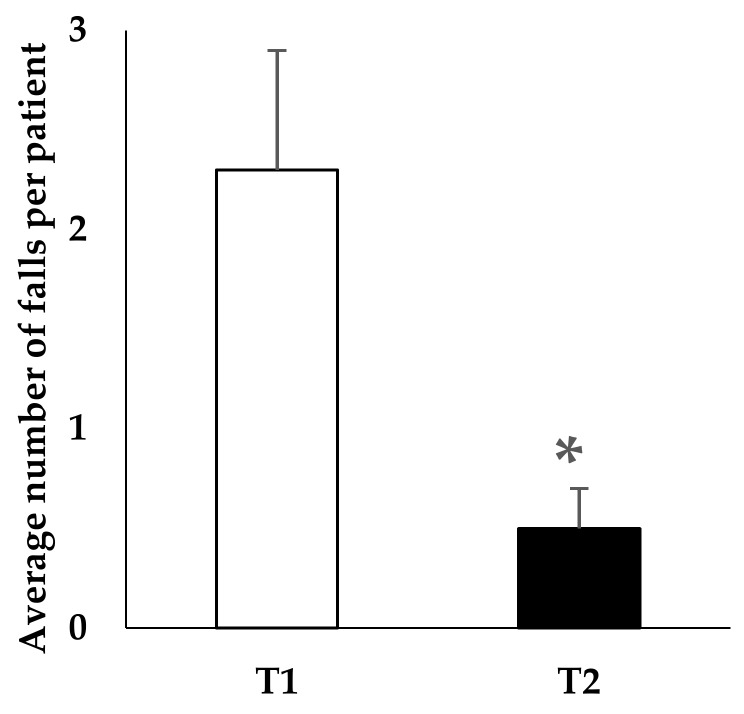
Benzodiazepines withdrawn population: mean number of falls per patient over a six-month period prior to the onset of withdrawal (T1) and within the same six-month period after the reassessment of prescriptions at one year (T2). Significant difference between T1 and T2 (* *p* = 0.01—Student’s *t*-tests on matched values). Study carried out in a French nursing home.

The analysis of the number of falls per patient in the T1 period before the start of the withdrawal program did not reveal a significant difference between the two groups of residents (future non-withdrawn (patients who continued to take BZD) and withdrawn patients in BZD): mean of 2.1 ± 1.3 and 2.3 ± 0.6 falls per patient, respectively. For the non-withdrawn patients in BZD, the number of falls per patient was not significantly different between T1 and T2 periods. Conversely, as shown in Figure 1, the number of falls was significantly decreased in the completely withdrawn population (patients with BZD arrest) between the T1 and T2 periods (2.3 ± 0.6 vs. 0.5 ± 0.2 falls, *p* = 0.01).

## 4. Discussion

The incentive program implemented in the nursing home identified 31 residents under BZD therapy and 11 accomplished total BZD withdrawal after one year. This withdrawal was accompanied by a significant reduction in the number of falls evaluated before and after the program for each resident.

Among BZDs with long half-life, four patients under clonazepam for anxiolysis were able to stop their treatment with a strategy of gradual reduction of doses, without appearance of withdrawal symptoms despite the difficult withdrawal of BZDs with long half-lives [16].

The simple “treatment diminution” (integrating the dosage decrease or the transition to a shorter half-life BZD) concerned two patients at the end of the one-year follow-up. This partial withdrawal finds its clinical legitimacy on the basis of data from Billioti de Gage et al. [17]. It could be optimized by psychological supports, but these approaches are associated with increased caregiver time and obviously a higher cost [18,19,20].

The absence of paroxetine prescription, a withdrawal option according to the HAS, was explained by the willingness of prescribers to avoid any potential iatrogenic overload in older subjects already having multiple prescriptions at the somatic level. This strategy could, however, find its legitimacy for non-withdrawn patients in our study who correspond to situations judged to be unfavorable according to the data of self- and/or hetero-evaluations.

In all cases, changes in prescription with treatment discontinuation or dosage decrease did not necessitate higher doses reintroduction in the year of follow-up. These elements indicate that the ease with which the BZD discontinuation was carried out was seen in the continual reduction in BZD dosages and not in higher BZD reintroduction without clinical symptoms requiring the interruption of the procedure.

Studies linking BZD to the potential risk of falls in the older people and their consequences are numerous in the international literature [4,5,6,7,10,11,21,22,23,24]. However, to our knowledge, no French study conducted in nursing homes has been correlated with a reduction in falls with a BZD withdrawal program. This lack of data in nursing homes is in line with the Verger report stating that “there is very little clinical research data on older adults living in long-term nursing homes” [25]. The increased risk of falling in elderly is regularly associated with long-acting benzodiazepines [4,7]. However, other data show that benzodiazepines with short elimination half-life and/or high dose levels of Z-drugs can also increase the risk of falls [5,11]. Interestingly, given the advanced age of our population (average age, 89 years), Pariente et al. noted that BZD use was significantly associated with the occurrence of fall-related injuries, with a significant interaction with age (adjusted odds ratio for injurious falls in patients under BZD was 2.2 (95% confidence interval [CI] 1.4, 3.4) in patients with an age greater than or equal to 80 years and 1.3 (95% CI 0.9, 1.9) in patients with an age lesser than 80 years) [6]. Moreover, in 2013, a cross-sectional study with data from the French national healthcare insurance system estimated that 7% and 13.4% of BZD users presented comorbidities at increased risk for falls or fractures for patients with an age lesser than 80 years and with an age greater than or equal to 80 years and, respectively [10].

Beyond falls, more recent current data on potential long-term disturbances of BZD reinforce the need to withdraw patients [9,16]. Billioti de Gage et al. [9] showed in 2012 that the use of BZD increases the risk of developing dementia by 50% compared to those who have never used it (Personnes âgées Quid-PAQUID study). In 2014, it was noted that there was a dose–effect relationship between BZD and Alzheimer’s disease in older people treated previously for more than three months, with the risk being higher for long acting formulations (>20 h in the study) [16]. Although the same team confirmed these data (with data of the Three-City Study (3C Study [26] and also see the recent review [27]), contradictory data exist and long follow-up duration applied in large prospective cohort studies appears to be required [28,29].

## 5. Conclusions

This study shows that the implementation of BZD withdrawal program allowed for 11 patients to achieve complete BZD cessation after one year. This data was achieved in gradual dose reduction. The example of this work may encourage nursing homes to consider such strategies whose benefit–risk ratio is highly favorable. Its immediate impact is visible in the prevention of falls, while its secondary impact on dementia evolution, based on literature data, may be considered, but remains to be explored.

## Figures and Tables

**Table 1:** Four-month and one-year evolution of benzodiazepine (BZD) deprescription practices in a nursing home for dependent older adults in France, following the implementation of a withdrawal program.

Starting the Withdrawal Program	Patient’s Follow-Up under BZD at Four Months	Patient’s Follow-Up under BZD at One Year
Number of patients under BZD *n* = 31 patients (28.7% of the patients in nursing home)	Total number of patients *n* = 31	Total number of patients *n* = 29
	15 patients: maintenance	8 patients: maintenance 1: diminution 2: stop 4: deceased
	7 patients: diminution	1 patient: diminution 6: stop
	7 patients: stop	3 patients: stop (maintenance) 4: deceased
	1 sight loss (transferred)	
	1 deceased	

## References

[1] Pelissolo A., Bisserbe J.C. (1994). [Dependence on benzodiazepines. Clinical and biological aspects]. L’Encephale.

[2] Bateson A.N. (2002). Basic Pharmacologic Mechanisms Involved in Benzodiazepine Tolerance and Withdrawal. Curr. Pharm. Des..

[3] Haute Autorité de Santé-Modalités d’arrêt des Benzodiazépines et Médicaments Apparentés chez le Patient Agé. https://www.has-sante.fr/portail/jcms/c_601509/fr/modalites-d-arret-des-benzodiazepines-et-medicaments-apparentes-chez-le-patient-age.

[4] Koski K., Luukinen H., Laippala P., Kivelä S.L. (1998). Risk factors for major injurious falls among the home-dwelling elderly by functional abilities. A prospective population-based study. Gerontology.

[5] Landi F., Onder G., Cesari M., Barillaro C., Russo A., Bernabei R. (2005). Silver Network Home Care Study Group Psychotropic medications and risk for falls among community-dwelling frail older people: An observational study. J. Gerontol. A Biol. Sci. Med. Sci..

[6] Pariente A., Dartigues J.-F., Benichou J., Letenneur L., Moore N., Fourrier-Réglat A. (2008). Benzodiazepines and injurious falls in community dwelling elders. Drugs Aging.

[7] Berdot S., Bertrand M., Dartigues J.-F., Fourrier A., Tavernier B., Ritchie K., Alpérovitch A. (2009). Inappropriate medication use and risk of falls—A prospective study in a large community-dwelling elderly cohort. BMC Geriatr..

[8] Barker M.J., Greenwood K.M., Jackson M., Crowe S.F. (2004). Persistence of cognitive effects after withdrawal from long-term benzodiazepine use: A meta-analysis. Arch. Clin. Neuropsychol. Off. J. Natl. Acad. Neuropsychol..

[9] Billioti de Gage S., Bégaud B., Bazin F., Verdoux H., Dartigues J.-F., Pérès K., Kurth T., Pariente A. (2012). Benzodiazepine use and risk of dementia: Prospective population based study. BMJ.

[10] Bénard-Laribière A., Noize P., Pambrun E., Bazin F., Verdoux H., Tournier M., Bégaud B., Pariente A. (2016). Comorbidities and concurrent medications increasing the risk of adverse drug reactions: Prevalence in French benzodiazepine users. Eur. J. Clin. Pharmacol..

[11] Yu N.W., Chen P.J., Tsai H.J., Huang C.W., Chiu Y.W., Tsay W.I., Hsu J., Chang C.M. (2017). Association of benzodiazepine and Z-drug use with the risk of hospitalisation for fall-related injuries among older people: A nationwide nested case-control study in Taiwan. BMC Geriatr..

[12] Briot M. (2006). Assemblée Nationale, Office Parlementaire d’Evaluation des Politiques de Santé (OPEPS). Rapport sur le Bon Usage des Médicaments Psychotropes. http://www.assemblee-nationale.fr/12/rap-off/i3187.asp.

[13] Mebarki S., Trivalle C. (2012). Échelles d’évaluation de l’effet anticholinergique des médicaments. NPG Neurol. Psychiatr. Gériatr..

[14] Rickels K., DeMartinis N., García-España F., Greenblatt D.J., Mandos L.A., Rynn M. (2000). Imipramine and buspirone in treatment of patients with generalized anxiety disorder who are discontinuing long-term benzodiazepine therapy. Am. J. Psychiatry.

[15] (2012). World Health Organization. http://www.who.int/mediacentre/factsheets/fs344/en/.

[16] AFSSAPS Report de mise en Application de la Restriction de Prescription Initiale Annuelle aux Neurologues et aux Pédiatres des formes orales de Rivotril^®^ (clonazépam). Lettre aux Professionnels de Santé (Report du 2 Janvier 2012 au 15 Mars 2012). Décembre 2011. http://ansm.sante.fr/Activites/Surveillance-des-stupefiants-et-des-psychotropes/Medicaments-a-risque-d-usage-detourne-ou-de-dependance/Medicaments-a-risque-d-usage-detourne-ou-de-dependance/RIVOTRIL.

[17] De Gage S.B., Moride Y., Ducruet T., Kurth T., Verdoux H., Tournier M., Pariente A., Bégaud B. (2014). Benzodiazepine use and risk of Alzheimer’s disease: Case-control study. BMJ.

[18] Vicens C., Socias I., Mateu C., Leiva A., Bejarano F., Sempere E., Basora J., Palop V., Mengual M., Beltran J.L. (2011). Comparative efficacy of two primary care interventions to assist withdrawal from long term benzodiazepine use: A protocol for a clustered, randomized clinical trial. BMC Fam. Pract..

[19] Voshaar R.C.O., Couvée J.E., van Balkom A.J.L.M., Mulder P.G.H., Zitman F.G. (2006). Strategies for discontinuing long-term benzodiazepine use: Meta-analysis. Br. J. Psychiatry J. Ment. Sci..

[20] Parr J.M., Kavanagh D.J., Cahill L., Mitchell G., McD Young R. (2009). Effectiveness of current treatment approaches for benzodiazepine discontinuation: A meta-analysis. Addict. Abingdon Engl..

[21] Pierfitte C., Macouillard G., Thicoïpe M., Chaslerie A., Pehourcq F., Aïssou M., Martinez B., Lagnaoui R., Fourrier A., Bégaud B. (2001). Benzodiazepines and hip fractures in elderly people: Case-control study. BMJ.

[22] Coutinho E.S.F., Fletcher A., Bloch K.V., Rodrigues L.C. (2008). Risk factors for falls with severe fracture in elderly people living in a middle-income country: A case control study. BMC Geriatr..

[23] Finkle W.D., Der J.S., Greenland S., Adams J.L., Ridgeway G., Blaschke T., Wang Z., Dell R.M., VanRiper K.B. (2011). Risk of fractures requiring hospitalization after an initial prescription for zolpidem, alprazolam, lorazepam, or diazepam in older adults. J. Am. Geriatr. Soc..

[24] Xing D., Ma X.L., Ma J.X., Wang J., Yang Y., Chen Y. (2014). Association between use of benzodiazepines and risk of fractures: A meta-analysis. Osteoporos. Int..

[25] Verger P. (2013). La Politique du Médicament en EHPAD.

[26] Shash D., Kurth T., Bertrand M., Dufouil C., Barberger-Gateau P., Berr C., Ritchie K., Dartigues J.F., Bégaud B., Alpérovitch A. (2016). Benzodiazepine, psychotropic medication, and dementia: A population-based cohort study. Alzheimers Dement..

[27] Billioti de Gage S., Pariente A., Bégaud B. (2015). Is there really a link between benzodiazepine use and the risk of dementia?. Expert Opin. Drug Saf..

[28] Gray S.L., Dublin S., Yu O., Walker R., Anderson M., Hubbard R.A., Crane P.K., Larson E.B. (2016). Benzodiazepine use and risk of incident dementia or cognitive decline: Prospective population based study. BMJ..

[29] Zhong G., Wang Y., Zhang Y., Zhao Y. (2015). Association between Benzodiazepine Use and Dementia: A Meta-Analysis. PLoS ONE.

